# Does Breast Cancer Increasingly Affect Younger Women?

**DOI:** 10.3390/ijerph17134884

**Published:** 2020-07-07

**Authors:** Katarzyna Konat-Bąska, Rafał Matkowski, Jerzy Błaszczyk, Dawid Błaszczyk, Urszula Staszek-Szewczyk, Natalia Piłat-Norkowska, Adam Maciejczyk

**Affiliations:** 1 Katedra Onkologii, Uniwersytet Medyczny im. Piastów Śląskich we Wrocławiu (Department of Oncology, Wroclaw Medical University), 53-413 Wrocław, Poland; konat.katarzyna@dco.com.pl (K.K.-B.); urszula.staszek-szewczyk@umed.wroc.pl (U.S.-S.); maciejczyk.a@dco.com.pl (A.M.); 2 Dolnośląskie Centrum Onkologii we Wrocławiu (Wroclaw Comprehensive Cancer Center), 53-413 Wroclaw, Poland; blaszczyk.jerzy@dco.com.pl (J.B.); blaszczyk.dawid@dco.com.pl (D.B.); nataliapilat12@gmail.com (N.P.-N.)

**Keywords:** breast cancer, young women, screening, incidence

## Abstract

Breast cancer is the most frequently diagnosed malignant neoplasm among females. The proportion of women diagnosed in the premenopausal period is relatively small. Nevertheless, this is the most commonly diagnosed cancer among young women. The aim of the study was to analyze the incidence rate of breast cancer in a group of young women based on data obtained in the Lower Silesian Voivodeship between 1984 and 2016. A total of 34,251 women with a diagnosis of invasive breast cancer were analyzed. The median age of diagnosis exhibited an upward trend from 57 to 63. The youngest age of breast cancer diagnosis did not decrease. Women up to the age of 24 were sporadically diagnosed. Given the total number of cases, the proportion of women under the age of 39 was approximately 5%, and it did not increase throughout the entire examination period. The major increase in the growth trend during the analyzed period was observed in a group of women aged of 50–69 (regression coefficient: +24.9) and above 70 (regression coefficient +21.2). In a group of women under 40 the regression coefficient was only +4. It seems that breast cancer does not increasingly affect younger women since the risk in this age group remains low. However, an increasing incidence rate of breast cancer is more commonly observed in premenopausal women.

## 1. Introduction

Breast cancer is the most frequently diagnosed malignant neoplasm among women both in Poland and worldwide. According to GLOBOCAN data, in 2018, approximately 2.1 million women were diagnosed with breast cancer, which constitutes 11.6% of all malignant neoplasms. It means that 1 out of 4 women with a neoplasm is currently suffering from breast cancer [1].

There were 18,529 newly diagnosed breast cancer cases in Poland in 2017, which constitutes 22.5% of all cancers cases among women [2,3]. 

This malignant neoplasm is the second most common cause of cancer-related deaths in the world, just after lung cancer (626,679 cases −6.6%) [1]. In comparison in Poland it was 16% in 2017 [2].

The incidence of malignant neoplasm of the breast is constantly on the rise [1,4]. The American Cancer Society reported that breast cancer incidence rate increased by 0.3% per year during 2012–2016 period [5]. They also estimate that in 2020, breast cancer will affect 276,480 women in the United States alone [6]. Over the last three decades, breast cancer incidence rate has more than doubled in Poland. The highest growth was observed in women aged 50–69. Breast cancer incidence rate in Poland in 2025 is estimated to increase by 50% compared to 2006 [4].

The proportion of women diagnosed with breast malignant neoplasm in the premenopausal age is relatively small. In 2019 all DCIS cases among women under 40 years constituted only 2% of all cases and invasive breast cancers constituted 4% of all age groups [7]. Nevertheless, as the American data shows, that breast cancer is the most commonly diagnosed cancer among women aged 20–49. It is also the main cause of death in the group of patients aged 30–49 [8]. In Poland, the situation is similar. According to the Polish National Cancer Registry, in the population of young women aged 20–44, breast cancer is the most common type of cancer, accounting for 27% of cases, and results in the highest number of deaths from oncological causes −29% [2].

Although age is a major risk factor for breast cancer, a number of recent studies have revealed an increase in the incidence rate of this type of cancer among premenopausal women [9,10,11,12,13,14].

With regard to the figures mentioned above, the increasing number of younger women suffering from breast cancer is becoming a public concern reported by the media. That is because the younger women are not in scope of breast cancer screening programs in Poland, which are primarily focused on women aged 50–69. Breast cancer among young women is also a serious psychosocial problem. Cancer diagnosis and oncological treatment may impact quality of life as it causes premature menopause and impaired fertility [15,16].

The aim of the study was to analyze trends in the incidence rate of breast cancer in the group of young women (<age of 40) based on data obtained in the Lower Silesian voivodeship covering the period from 1984–2016. 

## 2. Materials and Methods

The analysis involved data archived in the Cancer Registry of Wroclaw Comprehensive Cancer Center in 1984–2014 (30,808 cases) and data from the Polish National Cancer Registry of the Institute of Oncology in Warsaw in 2015–2016 (3443 cases) [3].

Since 1999, the territory of Poland has been divided into 16 voivodeships (before that time there were 49 voivodeships). Organizationally, a voivodeship is a counterpart of a country in the UK or a state in the USA. The average number of people in a voivodeship ranged from 982,000–5,423,000 in 2019. Population data for the Lower Silesian voivodeship in 5 year-long age groups has been available since 1999, when the voivodeship was established (until 1998 the term “Lower Silesia” included four voivodeships—Wrocławskie, Wałbrzyskie, Legnickie and Jeleniogórskie). Because of this, we do not have comparable information from the Central Statistical Office regarding the Lower Silesian Voivodeship’s population before year 1999.

The analyzed set was created on the basis of data collected on 12 November 2019.

A total of 34,251 women with the diagnosis of in situ and invasive breast carcinoma were analyzed: D05 and C50 diagnosis code according to ICD 10 classification. The data encompassed 31 years and was divided into seven periods: a six year-long period between 1984 and 1989, five year-long periods between 1990 and 2014 and a two year-long period between 2015 and 2016. In order to make the data comparable to national and global data, some five year-long periods have been combined to form ranges: up to 24, 25–39, 50–69 and over 70 years of age. Two five year-long periods remained in the analysis: 40–44 and 45–49.

The population of Poland by age subgroups was obtained from Statistics Poland (data as of 12 November 2019).

We further calculated Poisson regression modeling for a consecutive time period and for a successive age band. The age-specific incidence rate per 100,000 women from 1999 in the above-mentioned age groups and changes for trends in individual age groups with linear regression were calculated. Risk ratio, regression coefficient and *p*-value were determined.

Voivodeship and National Cancer Registry have a general authorization to collect and analyze anonymized data. Formal ethical approval and patient consent for this study was not required.

No information about ethnicity of population or genetic predisposition to breast cancer were available in the analyzed database.

## 3. Results

The median age of diagnosis in the time span from 1984 to 2009 was constant and ranged from 57 to 58 years of age. In subsequent years, it showed an upward trend. In the period 2010–2014, the median age of diagnosis increased by three years, and in 2015–2016 by another two years (Table 1). The age of the youngest woman diagnosed with breast cancer did not decrease in the selected periods. In the time span from 1984 to 1989, it was 17 years of age, and after, it was between 20 and 22 (Table 1). 

Women up to the age of 24 were sporadically diagnosed with breast cancer. During the analyzed 33 years, there were 45 such patients-on average one to two diagnoses per year. In the years 1984–1989, there were 9 patients (0.3%), and in 1990–1994 there were 3 patients (0.07%) (Table 2).

**Figure 1:**
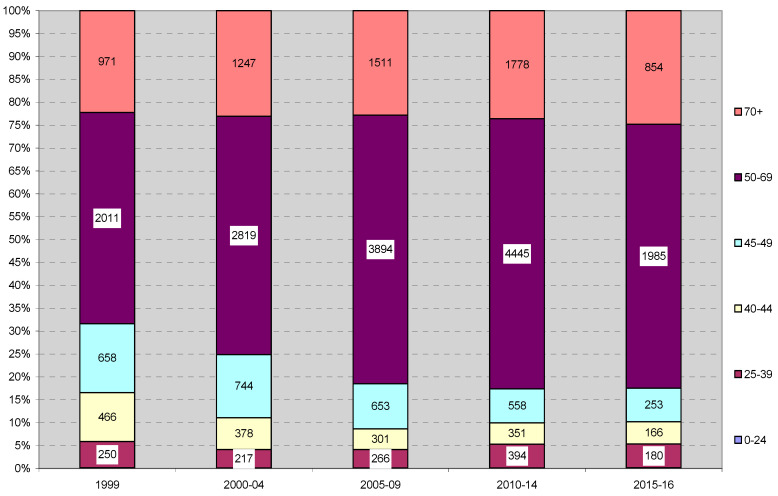
Age structure and number of malignant neoplasms of breast cases for selected age groups and periods.

To better illustrate changes, the percentage share of selected age groups was calculated in relation to all diagnosed cases and presented in the graph (Figure 1). The number of patients diagnosed with breast cancer increases considerably in the 50–69 age group and less in the 70+ age group. The remaining, younger age, groups decreased their share (40–49) or maintained a constant share (up to the age of 39). In the entire period, the incidence among women under the age of 40 was approximately 5% (from 12.5% between the years 1984–1989 to 4.1% between the years 2000–2009).

**Figure 2:**
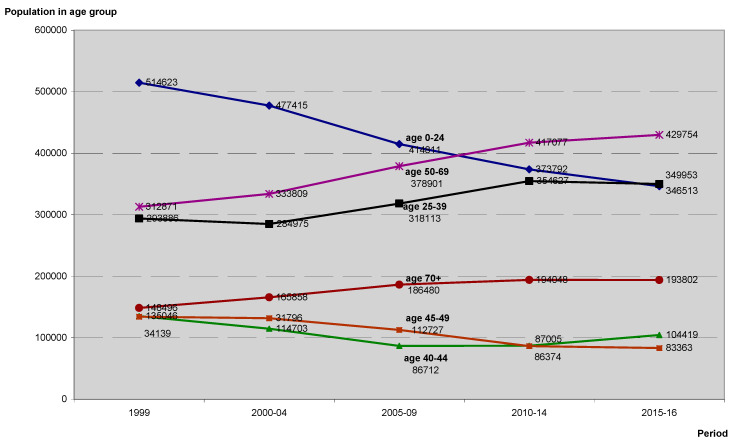
Changes in the average annual number of women in Lower Silesian voivodeship in the analyzed age groups according to the studied periods.

The graph shows that the population was ageing, with a rapid increase in the average number of women in the group with the highest incidence of breast cancer, aged 50–69 and 70+. The size of younger groups, with the exception of 25–39 years old, was decreasing (Figure 2).

Following the estimates of the Poisson regression modeling (Figure 1 and Figure 2), significant growth of the number of cancer cases was observed; for a consecutive time period, the estimated risk ratio RR = 1.13 [1.12, 1.14], *p* < 0.0001. Additionally, using the Poisson regression approach, the risk of cancer increased with patients’ age; for a successive age band, the predicted RR = 1.81 [1.79, 1.82], *p* < 0.0001.

**Figure 3:**
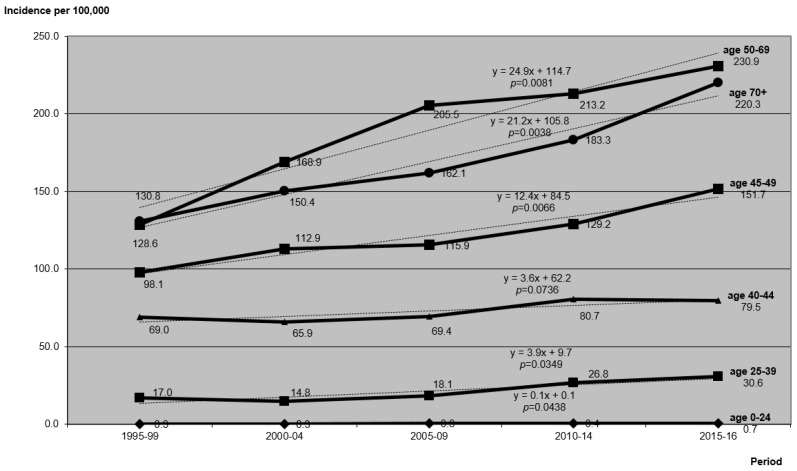
Changes and trends in the average annual age-specific incidence rates per 100,000 women in selected age groups and periods.

Breast cancer incidence rates increased in all age groups. The highest change between the analyzed periods was found in the group of women aged 50–69 (regression coefficient: +24.9; *p*-value: 0.0081) and above 70 years of age (regression coefficient: +21.2; *p*-value: 0.0038), then 45–49 (regression coefficient: +12.4; *p*-value: 0.0066). Among women between age of 40–44, increase was not statistically significant (*p*-value: 0.0736) with regression coefficient +3.6. In the group of women under 40, increase in incidence rate was statistically significant but not as sharp as in older age groups. Regression coefficient for age group 25–39 was +3.9 (*p*-value: 0.0349), and for age group 0–24 years, it was +0.1 (*p*-value: 0.0438) (Figure 3).

## 4. Discussion

The aim of this study was to analyze breast cancer incidence trends among young women defined as under the age of 40 in the Lower Silesian voivodeship. The results presented are consistent with national and global data. The incidence rate increases most rapidly in the group over 50 years of age, which is probably related to the use of screening mammography. In the group of women under 24 years of age, breast cancer diagnoses constitute individual cases. However, in the 0–39 age group, even though it is still a small percentage, there is a continuing upward trend in the incidence. The situation may be alarming as this group is not included in screening programs. As cancer diagnosed in the younger age groups tends to be at more advanced stages, and the subtypes with poorer chances of making a full recovery are also more often diagnosed, the social and economic impact can be quite significant. 

Data from the American population collected between 1992 and 2004 show an increase in breast cancer incidence among white women under 40 (annual percentage changes (APC): 0.47 [17]. A more recent study shows stable incidence rate in women aged 20–49 between 1999 and 2002 and an increase of about 0.5% per year between 2002 and 2015 [8]. Another study, where the 20–39 age group between 1975 and 2015 was analyzed, presents an increase in age-adjusted incidence from 24.6 per 100,000 in 1975 to 31.7 per 100,000 in 2015 (APC: 0.5) with two periods of significant changes: 1975–1984 APC: 2.3 and 1994–2015 APC: 0.7 [11]. 

In Canada, a substantial increase in the incidence rate among women aged 20–29 was observed between 1998 and 2015 (APC: 2.92), while in the 30–69 age group, the incidence rate did not change significantly during the analyzed time period [12]. 

Data collected by 17 European cancer registries covering the 1995–2006 period show a steady increase in annual incidence rates of cancer in the 20–39 age group. The increase in the 20–29 age group was greater and amounted to 3.2%, while in the 30–39 age group it was 1.4% [9]. 

The Leclere and GRELL cooperative study also presented data from several European countries. They involved patients under the age of 40 who were diagnosed with breast cancer in the years 1990–2008. Of the diagnoses, 63.5% were made in the 35–39 age group. The incidence rate increased linearly on average by 1.19% per year. Differences were observed depending on the country: from 0.2% in Bulgaria to 2.68% in Portugal. The increase in the incidence rate was greater for the group: 15–34 years of age (APC: 2.0) compared to the group: 35–39 years of age (APC: 1.1) [13].

Spanish data from the 1980–2004 period show a steady increase of 1.7% per year in the incidence rate among women aged 25–44 [18]. A more recent study performed between 1985 and 2012, covering all age ranges, shows the greatest increase in the incidence rate among women under 40 (APC: 3.5). The analysis also revealed a decrease in mortality in all age groups, the highest in the 50–69 age group. Interestingly, no relationship has been established between the decline in mortality and the implementation of screening programs. Introduction of the screening increased the incidence of stage I tumors, with no decrease in the incidence of more advanced stages [14]. 

In the Swiss population, among women diagnosed with breast cancer between 1995–2004, patients aged 25–39 accounted for 3.4% in 1995 and 7.2% in 2004. In the 25–39 age group, breast cancer incidence rate was stable until 2002, after which it began to increase from 19.7/100,000 cases in 1995 to 53.9/100,000 in 2004. The average annual growth rate in the analyzed period was 8.7%, of which in the last 3 years, 2002–2004, it amounted to 46.7% annually [19]. Another study analyzed women aged 20–49 years, in the years 1996–2009, revealing an increased incidence rate from 57.4 per 100,000 in 1996 to 68.3 per 100,000 in 2009 (APC of 0.8%) with the largest increase in the younger age group 20–39 years (APC 1.8%) [10]. 

Breast malignant neoplasms in women of different age groups are not homogenous. Lesions diagnosed in the premenopausal age differ significantly in etiology and morphology from those observed in older women. Among the younger age group, breast cancer is generally more aggressive: There are fewer preinvasive lesions, and invasive carcinomas are diagnosed more often in a larger tumor size, higher stage and with positive nodes. Moreover, neoplasms with negative estrogen and progesterone receptors, higher expression of human epidermal growth factor 2 (**HER2)** and with more poorly differentiated histologic grade are diagnosed more frequently. Additionally, in the younger patients group, percentage of gene mutations predisposing to breast cancer occurrence, such as breast cancer genes 1 and 2 (**BRCA1/2**), is higher. Local and regional relapses are more frequent, and the risk of distant metastases and death is higher, which is reflected in a lower 5-year survival rate [20,21,22,23,24,25]. 

Increased risk of developing the disease is influenced by many factors. The main one is older age, followed by socio-economic status, genetic factors (carrying mutated **BRCA1** or **BRCA2** genes), positive family history, exposure to sex hormones (early menarche, late menopause, older age at first birth, long-term hormone replacement therapy or hormonal contraception), overweight and obesity and exposure to ionizing radiation (chest radiotherapy before the age of 30) [20,26]. A recent study of American population suggests that increased BMI is associated with a decrease in breast cancer incidence rate among younger women. Lower fertility rates result in higher breast cancer incidence rates [27]. Black women have significantly higher breast cancer incidence rates among females with breast cancer aged <45 years [28].

Studies show worse treatment results among women in younger age groups. The 5- and 10-year survival rates account for 89% and 80% for all breast cancer patients, respectively, while the same rates are 82% and 71% for patients under 35 years of age. Patients under 35 years of age with ER-negative cancer have the worst chances of making a full recovery [29].

Although treatment results in the younger age group are worse than in older patients, the improvement in curability observed in recent years is also noticeable in this group of patients.

Comparing the periods 1990–1994, 1995–1999 and 2000–2004 for patients aged 40–50 with ER-positive cancer, there is a decrease in mortality of 23% and 35%, respectively. For an analogous group of ER-negative cancer patients, the mortality rate was 2% and 10%, respectively. Mortality rates were also decreasing in patients under the age of 40 [29]. In his study, Guo analyzed the results of treatment of women aged 20–39 between 1975 and 2015, showing a 60–70% decrease in mortality over the studied 4 decades. The improvement in treatment results was visible in the increase in progression-free survival rates not only in the group of women with locally advanced cancer but also in the group of women with metastatic disease, both in the group of women <40 as well as in the older age groups [11]. 

The improvement in treatment results in premenopausal patients does not result from implemented screening programs, as patients of that age are not normally covered by them. In addition, when comparing different age ranges for the introduction of screening programs in European countries, a similar reduction in mortality is observed. This leads to the conclusion that it is not screening that improves survival but the progress that has been made in treatment regimens and techniques. Reduction of 15-year cancer–specific mortality risk is higher in patients under the age of 50; after chemotherapy, it is 38% vs. 20%; if hormonal therapy is included, the reduction of mortality risk is 57 vs. 45 in patients under and over 50 years of age. Studies confirm that the results can be attributed to the improvement of both local treatment techniques such as surgery and radiotherapy as well as systemic therapy: hormonal therapy and chemotherapy [30,31].

It is a common knowledge that early diagnosis and treatment significantly contribute cancers’ curability. In young women without risk factors, MMG is not recommended. There are no randomized studies available that would indicate a decrease in mortality after the implementation of this examination on a routine basis in the aforementioned age group. In addition, mammograms are less sensitive in younger women due to increased breast density. Positive predictive value of MMG among women aged 35 to 39 is only about 1.3% [32]. Additionally, MMG involves exposure to ionizing radiation, which translates to a higher risk of radiation-induced secondary cancer among young patients [33]. Other imaging examinations such as ultrasound and MRI seem to be more effective in diagnosing suspicious lesions in young women [34,35,36].

Guidelines recommend that the above examinations should be performed in the form of screening tests for patients with a higher risk of developing breast cancer at an early age [37,38]. This group includes patients with a diagnosed **BRCA1/2** mutation, a positive family history or chest irradiation at a young age. 

Analyzed age group comprises patients of reproductive age. Neoplasms during pregnancy are diagnosed very rarely (0.02–0.1% of all pregnancies). The most frequently diagnosed neoplasm in this group of women is breast cancer (36%). In 2018, a panel of experts from the Polish Society of Surgical Oncology, the Polish Society of Clinical Oncology and the Polish Society of Gynecologists and Obstetricians developed a common position on the performance of breast ultrasound examination in pregnant women. According to it, it is recommended for pregnant women over the age of 35 to perform not only a standard clinical breast examination by a gynecologist-obstetrician but also breast ultrasound during the first or second trimester of pregnancy. In pregnant women under the age of 35, with no symptoms of breast disease and no family/genetic history of breast cancer, breast ultrasound should be considered in the first or second trimester of pregnancy [39].

The authors are aware of the limitations of this study. Only Lower Silesian population was analyzed. Additionally, we do not have information about ethnicity, genetic predisposition or any other breast cancer risk factors in our database. Moreover, the authors did not analyze in this study the impact of population screening introduced in Poland in 2007 for women aged 50 to 69. A strong point of this long-term study is that it covers the entire population of the region, which is around 1.5 million women.

## 5. Conclusions

The results of the above analysis indicate that it is not true that more and more young women are diagnosed with breast cancer more frequently since the risk remains low. The median age at which breast cancer is diagnosed increased from 57 to 63 years of age. Cases diagnosed in patients under the age of 24 are still sporadic: one to two per year. The number of cases among women over 50 increased the fastest. The proportion of women under the age of 39 in the total number of cases did not increase and throughout the whole period amounted to approximately 5%. Since 2010, the number of diagnosed cases in this group has significantly increased to 80 and 92 per year, whereas in the period 1984–2009 there were 45–60 cases per year. The change in the growth trend between the analyzed periods in this age group was +4. Therefore it is true that the commonly observed increase in the incidence rate of breast cancer also affects the group of women under 40 years of age.

Studies mention greater awareness, better access to examinations and, above all, increased exposure to risk factors related to procreation trends, contraception or diet and lifestyle as the reasons for such a situation. There are no studies confirming the necessity to introduce screening tests for a younger group of women. In women under 40 years of age, experts recommend regular screening only in a subgroup of women with an increased risk of breast cancer. 

## Figures and Tables

**Table 1:** Median age for malignant neoplasm of breast cases in 1984–2016.

	1984–1989	1990–1994	1995–1999	2000–2004	2005–2009	2010–2014	2015–2016
Median age	57	58	58	57	58	61	63
Age distribution	17–98	22–98	21–98	20–97	21–99	21–101	21–99
Number	2763	4363	3478	5411	6632	7536	3443

**Table 2:** Numbers of malignant neoplasm of breast cases in women under the age of 40.

Age Group	1984–1989	1990–1994	1995–1999	2000–2004	2005–2009	2010–2014	2015–2016
Total number of breast cancer cases	2763	4363	3478	5411	6632	7536	3443
Cases <24 years of age	9	3	7	6	7	8	5
Percentage of cases <24 years of age [%]	0.3	0.07	0.2	0.1	0.1	0.1	0.1
Cases <40 years of age	346	297	257	223	273	402	185
Annual average <40 years of age	58	60	51	45	55	80	92
Percentage of cases <40 years of age [%]	12.5	6.8	7.4	4.1	4.1	5.3	5.4
